# Establishing a Cohort of Transgender Men and Gender Nonconforming Individuals to Understand the Molecular Impact of Testosterone on Breast Physiology

**DOI:** 10.1089/trgh.2019.0040

**Published:** 2019-11-19

**Authors:** Gabrielle M. Baker, Michael E. Pyle, Adam M. Tobias, Richard A. Bartlett, Jordana Phillips, Valerie J. Fein-Zachary, Gerburg M. Wulf, Yujing J. Heng

**Affiliations:** ^1^ Department of Pathology, Beth Israel Deaconess Medical Center, Harvard Medical School, Boston, Massachusetts.; ^2^ Department of Plastic Surgery, Beth Israel Deaconess Medical Center, Harvard Medical School, Boston, Massachusetts.; ^3^ Division of Breast Imaging, Department of Radiology, Beth Israel Deaconess Medical Center, Harvard Medical School, Boston, Massachusetts.; ^4^ Division of Hematology/Oncology, Department of Medicine, Beth Israel Deaconess Medical Center, Harvard Medical School, Boston, Massachusetts.

**Keywords:** breast, gender dysphoria, plastic surgery, top surgery

## Abstract

**Purpose:** To characterize a cohort of transgender men and masculine-centered gender nonconforming individuals who underwent gender-affirming chest-contouring surgeries at our institution between 2013 and 2018.

**Methods:** Demographics, medical history, and breast histopathological assessment for 340 patients were retrieved from medical records.

**Results:** Most of our patients were white, non-Hispanic (75.0%), were taking testosterone (83.2%), and opted for chest-contouring surgery after 12–14 months of testosterone therapy. Ten patients were parous (2.9%). Seventy-nine (23.2%) and 27 (7.9%) patients had a family history of breast cancer or ovarian cancer, respectively. One transgender man was incidentally diagnosed with ductal carcinoma *in situ* at chest-contouring surgery.

**Conclusion:** Future studies on this cohort will provide valuable insights about the impact of testosterone on breast physiology.

## Background

The proportion of transgender adults in the United States has increased for the past decade.^1^ With increasing cultural and social acceptance, and insurance access, more transgender individuals are undergoing gender-affirming hormone therapy and/or surgery to alleviate their gender dysphoria. Specifically, transgender men (TM; trans men or female-to-male) and masculine-centered gender nonconforming individuals (GNCIs) are natal females who pursue testosterone therapy and/or gender-affirming surgery to enhance masculinization.^2^ Gender-affirming surgery for TM and GNCIs may include chest contouring, hysterectomy with or without bilateral salpingo-oophorectomy, creation of male genital prostheses, and facial esthetic procedures.^3^ The surgical removal of breast tissue is the most common first surgery for TM and GNCIs.^4^ Unlike mastectomy for oncological or breast cancer risk-reducing purposes, chest-contouring surgery in TM and GNCIs emphasizes an esthetic outcome and does not necessarily remove all mammary tissue.^5^

Testosterone therapy is a long-term process and its regimen follows the principle of hormone replacement therapy for cisgender men. The physiological testosterone range for cisgender men is 10-fold higher than cisgender women (300–1000 ng/dL vs. 30–100 ng/dL).^6^ Thus, TM and GNCIs use high-dose injectable testosterone esters (50–200 mg/week), transdermal testosterone gel (2.5–10 g/day), or transdermal testosterone patch (2.5–7.5 mg/day) to achieve testosterone levels comparable with cisgender men.^6^ These testosterone doses are >10-fold higher than the recommended treatment for hypoactive sexual desire disorder in cisgender women (300 μg/day).^7^ In other words, the amount of exogenous testosterone taken by TM and GNCIs is significantly higher than what is typically taken by cisgender women. Such long-term high-dose exposure to exogenous testosterone in TM and GNCIs raises concerns about the effect of testosterone therapy on their health.

Although testosterone therapy is generally considered safe,^8^ data are limited and there remains a knowledge gap about the impact of exogenous testosterone on hormone-sensitive tissues and hormone-dependent malignancies, for example, the breast and breast cancer. Surgical breast specimens from TM and GNCIs are a valuable resource to better understand the mechanism of action of long-term high-dose testosterone on breast physiology. This report describes our Transgender and Testosterone Therapy use (i.e., “Triple T”) research cohort consisting of TM and GNCIs with available archival breast tissue and histopathological slides at our institution between 2013 and 2018.

## Methods

### Subjects

This retrospective study was approved by the Beth Israel Deaconess Medical Center (BIDMC) Committee for Clinical Investigations (Protocol Number: 2018P000814). A total of 340 patients with gender dysphoria who underwent chest-contouring surgery between January 2013 and December 2018 at BIDMC, Boston, MA, were identified using surgical case lists from two plastic surgeons who perform the vast majority of chest-contouring surgeries at our institution.

### Data collection

Patient data from presurgical consult questionnaires, referral letters, and postsurgery follow-up were retrieved through electronic medical records. The electronic medical records were accessed between February 2019 and June 2019. Presurgical data include self-identified gender, home state, race/ethnicity, accompanying support person during consult, length and type of testosterone therapy, hormonal birth control use, body mass index (BMI), family history of breast and ovarian cancer, medical history including hysterectomy, reproductive history, smoking status, and alcohol consumption. Postsurgery data include age at surgery, breast histopathological findings, and updated information about hysterectomy and/or bilateral salpingo-oophorectomy. Length of testosterone use at surgery accounts for the additional time between presurgical consult and date of surgery, and reflects the duration of breast tissue exposed to testosterone.

### Analysis

Comparisons between two groups of patients who did and did not take testosterone therapy were analyzed using Mann–Whitney *U* test for continuous variables and Fisher's test for categorical variables. All analyses were conducted using R version 3.4.0 and *p*<0.05 was considered statistically significant.

## Results

Table 1 displays the demographics of our Triple T research cohort. The number of chest-contouring surgeries at BIDMC increased from 1 to 112 cases per year between 2013 and 2018. Our geographically diverse patients were from 15 U.S. states (CO, CT, MA, ME, NC, NH, NJ, NY, OH, PA, RI, TN, TX, VA, and VT) and 83.8% of them reside in MA. Most of our patients identified as TM (77.9%), 10.9% identified as GNCI, and 11.2% did not self-identify. The vast majority of our patients were white, non-Hispanic (75.0%). Thirty-six patients (10.6%) had hysterectomy and/or bilateral salpingo-oophorectomy after chest-contouring surgery compared with 26 (7.6%) who had the same procedure before chest-contouring surgery; 278 (81.8%) had not pursued this procedure at the time of medical record review. One patient had a previous chest wall reconstruction and six had previous breast reduction surgeries.

**Table 1.** Demographics of the 340 Transgender Men and Gender Nonconforming Individuals Who Had Chest-Contouring Surgeries at Beth Israel Deaconess Medical Center Between January 2013 and December 2018

	*n* (%)
Year of surgery	
2013	1 (0.3)
2014	7 (2.1)
2015	40 (11.8)
2016	75 (22.1)
2017	105 (30.9)
2018	112 (32.9)
Gender as identified by patient	
Trans male	265 (77.9)
Nonconforming	37 (10.9)
Not specified	38 (11.2)
Race and ethnicity	
White, non-Hispanic	255 (75.0)
White, Hispanic	7 (2.1)
Black or African American, non-Hispanic	28 (8.2)
Black or African American, Hispanic	2 (0.6)
Asian	11 (3.2)
Native American/Pacific Islander	3 (0.9)
Mixed race, non-Hispanic	8 (2.4)
Unspecified race and ethnicity	26 (7.6)
Companion at presurgery consult	
One or both parents	25 (7.4)
Other family member	3 (0.9)
Romantic partner	42 (12.4)
Spouse	3 (0.9)
Friend	4 (1.2)
Unknown	263 (77.4)
Hysterectomy and/or salpingo-oophorectomy	
Yes, before chest contouring	26 (7.6)
Yes, after chest contouring	36 (10.6)
No	278 (81.8)

Among 340 patients, 283 (83.2%) reported taking testosterone therapy before chest-contouring surgery compared with 55 (16.2%) who did not (Table 2). Testosterone use was not recorded for 2 patients (0.6%). The most frequent testosterone formulation was intramuscular testosterone enanthate/cypionate (249/283; 88.0%), followed by transdermal gel (14/283; 4.9%), transdermal patch (5/283; 1.8%), subcutaneous pellet (3/283; 1.1%), and transdermal cream (1/283; 0.4%). Testosterone formulation for 11 patients was unknown (3.9%). Most patients (38.9%) opt for chest-contouring surgery after pursuing testosterone therapy between 12 and 24 months (Table 2).

**Table 2.** Clinical Characteristics and Breast Cancer Risk Factors of the 340 Patients, Stratified by Testosterone Therapy Use

	All patients	Testosterone use	No testosterone use	p^*^
*n* (%)	340 (100.0)	283 (83.2)	55 (16.2)	
Age at surgery (median [IQR])	25.5 [22.0, 30.0]	25.0 [21.0, 30.0]	28.0 [24.0, 32.5]	0.09
Length of testosterone therapy at the time of chest-contouring surgery, *n* (%)				—
<1 year	62 (18.2)	62 (21.9)	—	
≥1 to <2 years	110 (32.4)	110 (38.9)	—	
≥2 to <5 years	66 (19.4)	66 (23.3)	—	
≥5 years	14 (4.1)	14 (4.9)	—	
Current user, unknown duration	31 (9.1)	31 (11.0)	—	
Never use	55 (16.2)	—	55 (100.0)	
Unknown	2 (0.6)	—	—	
Reproductive and hormonal factors				
Age of menarche (median [IQR])	12.5 [11.0, 13.0]	12.0 [11.0, 13.0]	13.0 [12.0, 13.5]	0.97
Parity, *n* (%)				0.46
Have children	10 (2.9)	7 (2.5)	3 (5.5)	
No children	148 (43.5)	124 (43.8)	24 (43.6)	
Unknown	182 (53.5)	152 (53.7)	28 (50.9)	
Hormone birth control use, *n* (%)				**<0.01**
Yes	16 (4.7)	8 (2.8)	8 (14.5)	
No	324 (95.3)	275 (97.2)	47 (85.5)	
Previous benign breast disease, *n* (%)				
Yes	12 (3.5)	9 (3.2)	3 (5.5)	0.42
No	328 (96.5)	274 (96.8)	52 (94.5)	
Family history				
Family history of breast cancer, *n* (%)				0.22
Yes, first degree	8 (2.4)	6 (2.1)	2 (3.6)	
Yes, second degree	47 (13.8)	41 (14.5)	6 (10.9)	
Yes, unknown degree	24 (7.1)	20 (7.1)	3 (5.5)	
No	202 (59.4)	173 (61.1)	29 (52.7)	
Unknown	59 (17.4)	43 (15.2)	15 (27.3)	
Family history of ovarian cancer, *n* (%)				0.16
Yes	27 (7.9)	24 (8.5)	3 (5.5)	
No	234 (68.8)	200 (70.7)	34 (61.8)	
Unknown	79 (23.2)	59 (20.8)	18 (32.7)	
Modifiable risk factors				
BMI at surgery (median [IQR])	26.1 [23.5, 30.4]	26.4 [23.7, 30.5]	25.1 [22.0, 29.6]	0.54
Alcohol consumption, *n* (%)				**0.03**
Current	188 (55.3)	148 (52.3)	39 (70.9)	
Never	142 (41.8)	126 (44.5)	16 (29.1)	
Unknown	10 (2.9)	9 (3.2)	0 (0.0)	
Tobacco smoker, *n* (%)				0.72
Current	23 (6.8)	21 (7.4)	2 (3.6)	
Ever	32 (9.4)	25 (8.8)	6 (10.9)	
Never	281 (82.6)	233 (82.3)	47 (85.5)	
Unknown	4 (1.2)	4 (1.4)	0 (0.0)	

^*^ *p* values were obtained using Mann–Whitney *U* or Fisher's test to compare between patients who did and did not use testosterone.

Bold indicates *p* values that are <0.05.

BMI, body mass index; IQR, interquartile range.

Of the available nonmodifiable breast cancer risk factors, 10 (2.9%) of our patients reported parity, 79 (23.2%) indicated a family history of breast cancer and 27 (7.9%) indicated a family history of ovarian cancer (Table 2). Twelve (3.5%) had prior benign breast disease: four were fibroadenomas, one was a cyst, and the histological subtype of the other seven cases were not recorded. Most of our patients did not use hormonal birth control (95.3%). The majority of them consumed alcohol (55.3%; Table 2). The average BMI of our Triple T cohort was 26.1.

Detailed histopathological review of surgical specimens of a subset of 115 patients have been conducted and reported elsewhere.^9^ Four of these 115 patients had atypical hyperplasia: 2 had atypical ductal hyperplasia (ADH), 1 had atypical lobular hyperplasia (ALH), and 1 had both ADH and ALH. Atypical hyperplasia is a proliferative histological subtype of benign breast disease that is known to increase subsequent breast cancer risk.^10,11^ One TM, 29 years old, was incidentally diagnosed with ductal carcinoma *in situ* at chest-contouring surgery. They had been taking testosterone cypionate 80 mg/week for 4 years, and their breast cancer 23-genes screening panel was negative, including negative for *BRCA* mutation.^5^

## Discussion

As the number of transgender adults increases, more research is critically needed to understand the impact of gender-affirming hormonal therapy and/or surgery on the health outcomes in this historically discriminated population. The impact of exogenous testosterone on hormone-dependent malignancies, such as breast cancer, in these individuals remains unclear.^12^ In this report, we described our Triple T cohort that consisted of 340 TM and GNCIs who had chest-contouring surgeries at our institution from 2013 to 2018. We collected clinical data, including testosterone therapy details, archival breast tissue blocks, and histopathology slides, to design future tissue-based studies to elucidate the molecular influence of testosterone on breast physiology and breast cancer risk in TM and GNCIs. Our future study will be valuable in supporting the health management of TM and GNCIs who pursue testosterone therapy.

Three population-based studies concluded that breast cancer risk in TM is lower than cisgender women.^13–15^ However, these studies only reported a limited number of 12 cases of breast cancer among 3698 TM and none of these studies characterized the genetic risk of those individuals. We categorized several important factors related to breast cancer risk in our cohort: BMI,^16^ reproductive factors,^16^ family history,^17^ previous benign breast disease,^18^ alcohol consumption,^19^ and tobacco smoking.^20^ It is important to note that these factors are associated with increased breast cancer risk in cisgender women. Breast cancer risk factors for TM and GNCIs have yet to be established. Based on our limited cohort numbers, these breast cancer risk factors for cisgender women do not appear to be different between TM and GNCIs who do and do not take testosterone therapy. More long-term follow-up studies are needed to identify factors that modulate breast cancer risks unique to TM and GNCIs. The histopathological findings of atypical hyperplasia and DCIS in a subset of our surgical specimens reiterates the need to establish culturally sensitive breast cancer screening protocols for TM and GNCIs, especially for those who do not undergo chest-contouring surgery.

The interplay between endogenous estrogens and exogenous testosterone in the breast remains poorly understood.^21^ Under conditions of excess, testosterone can be partially aromatized to estradiol and may drive breast cancer development.^22^ Indeed, higher plasma testosterone levels are associated with increased breast cancer risk in both pre- and postmenopausal cisgender women.^23,24^ Bentz et al. conducted the first and only molecular study in TM with five paired breast biopsies taken before and after 2 years of testosterone therapy.^25^ They reported that testosterone therapy was associated with a breast cancer gene signature and testosterone may initiate breast carcinogenesis.^25^ Therefore, larger observational studies are critically needed to assess how testosterone affects breast tissue health in TM and GNCIs. Neither testosterone use nor minimum length of testosterone use is mandatory for chest-contouring surgery. Thus, patients who did not use testosterone are ideal controls for subsequent research studies.

One challenge in conducting research to understand the molecular influence of testosterone on breast tissues is the unknown cumulative testosterone exposure and the lack of biomarkers to approximate androgen levels in the breast. Throughout the duration of therapy, an individual can interchange testosterone formulations (due to costs, availability, or personal preference), adjust testosterone dosage to optimize well-being, or pause therapy to conceive. Optimal testosterone dosing is not determined solely by serum levels but also by the patient's desire and whether their desired masculinizing effect is achieved. No study has systematically compared testosterone serum levels and the levels of testosterone and 5α-dihydrotestosterone in breast tissues. One potential biomarker could be the evaluation of androgen receptor expression as a surrogate to reflect androgen levels in the breast.^26–28^

The implementation of Section 1557 of the Affordable Care Act (ACA), signed into law in May 2016,^29^ partially explains the rapid increase in the number of chest-contouring surgeries conducted at BIDMC in 2017 and 2018. As expected, most of our patients underwent chest-contouring surgery before hysterectomy and/or bilateral salpingo-oophorectomy.^4^ With increasing cultural and social acceptance, and steadily improving health insurance policy coverage (i.e., access to better health care),^30^ we expect more TM and GNCIs to pursue chest-contouring surgeries at BIDMC, thus substantially increasing the numbers of our Triple T cohort in the next few years. As our cohort grows, we will continuously improve and update our questionnaires to capture valuable patient data, for example, how long they have been using a specific testosterone formulation. We could also perform blood work at time of presurgical workup, and collect fresh frozen breast tissues at the time of surgery.

## Abbreviations Used

ADH: atypical ductal hyperplasia
ALH: atypical lobular hyperplasia
BIDMC: Beth Israel Deaconess Medical Center
BMI: body mass index
GNCI: gender nonconforming individual
TM: transgender men
